# Caffeine as a Factor Influencing the Functioning of the Human Body—Friend or Foe?

**DOI:** 10.3390/nu13093088

**Published:** 2021-09-02

**Authors:** Kamil Rodak, Izabela Kokot, Ewa Maria Kratz

**Affiliations:** 1 Student Research Club, “Biomarkers in Medical Diagnostics”, Department of Laboratory Diagnostics, Division of Laboratory Diagnostics, Faculty of Pharmacy, Wroclaw Medical University, Borowska Street 211A, 50-556 Wroclaw, Poland; 2 Department of Laboratory Diagnostics, Division of Laboratory Diagnostics, Faculty of Pharmacy, Wroclaw Medical University, Borowska Street 211A, 50-556 Wroclaw, Poland; izabela.kokot@umed.wroc.pl

**Keywords:** caffeine, human body, caffeine action, oxidative-antioxidant balance

## Abstract

Nowadays, caffeine is one of the most commonly consumed substances, which presents in many plants and products. It has both positive and negative effects on the human body, and its activity concerns a variety of systems including the central nervous system, immune system, digestive system, respiratory system, urinary tract, etc. These effects are dependent on quantity, the type of product in which caffeine is contained, and also on the individual differences among people (sex, age, diet etc.). The main aim of this review was to collect, present, and analyze the available information including the latest discoveries on the impact of caffeine on human health and the functioning of human body systems, taking into account the role of caffeine in individual disease entities. We present both the positive and negative sides of caffeine consumption and the healing properties of this purine alkaloid in diseases such as asthma, Parkinson’s disease, and others, not forgetting about the negative effects of excess caffeine (e.g., in people with hypertension, children, adolescents, and the elderly). In summary, we can conclude, however, that caffeine has a multi-directional influence on various organs of the human body, and because of its anti-oxidative properties, it was, and still is, an interesting topic for research studies including those aimed at developing new therapeutic strategies.

## 1. Introduction

Caffeine is one of the most popular and widely consumed beverages in the world. Its main source is coffee, however, it may also be present in other plants such as tea leaves, guarana berries, and cacao beans. It is worth pointing out that caffeine may also be found in energy drinks, soft drinks, gums, and medications [1,2]. Chawla et al. [3] stated that average caffeine consumption from all sources reaches 76 mg/person/day; in the United States and Canada, it is approximately 210–238 mg/person/day and exceeds 400 mg/person/day in Sweden and Finland. On the other hand, Conway [4] reported that in 2020–2021, coffee consumption was around 166.63 million 60 kg bags worldwide. This huge interest in coffee is due to its taste attributes and stimulating effects, and it additionally stems from the culture and habits of people in many countries. Due to the times and culture we live in, the presence of coffee rituals in our daily lives does not seem particularly surprising. However, it is amazing that a humble Yemeni bush has wrapped its branches around all continents. Interestingly, despite such a large consumption of caffeine, research shows that it is more a habit than a compulsive addiction [5]. Recently, caffeine has been of scientific interest because, as a bioactive molecule, it has been shown to have beneficial health effects (e.g., it is able to protect against oxidative stress in Alzheimer’s disease (AD)) [6]. However, people with hypertension, children, adolescents, and the elderly may be more susceptible to the negative effects of caffeine consumption [7]. Due to the widespread presence of caffeine, we present its impact (both positive and negative) on the human body, taking into account individual systems.

### 1.1. Caffeine—General Information

**Figure 1:**
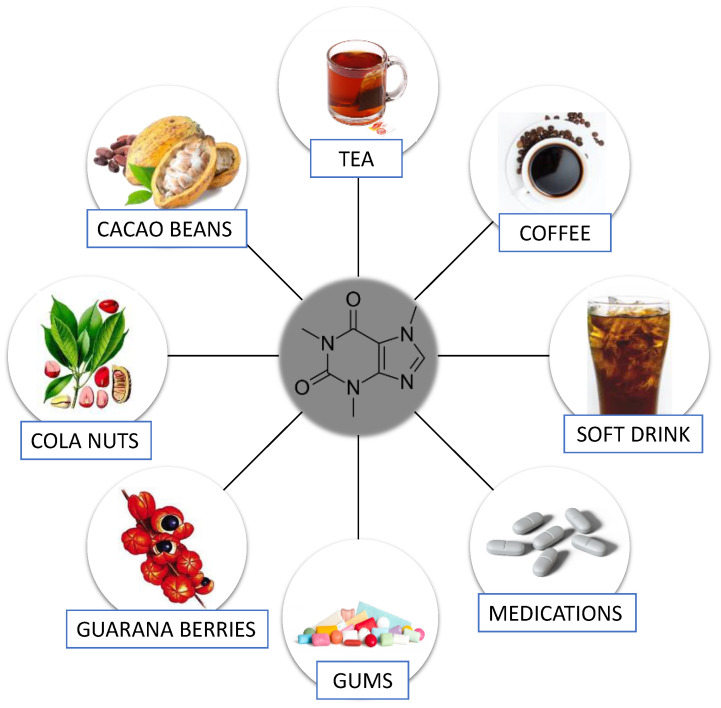
The most popular plants and products containing caffeine. Based on de Mejia et al. [10].

Caffeine (1,3,7-trimethylcanthine or 3,7-dihydro-1,3,7-trimethyl-1H-purine-2,6-dione), a well-known purine alkaloid, was described by Gennaro [8] as a white, odorless powder with a slightly bitter taste. Its chemical formula is C_8_H_10_N_4_O_2_. Caffeine occurs in more than 60 plant species globally [9]. This substance is produced by extraction from green coffee beans, tea leaves, and cola nuts, and also by synthetic procedures (e.g., methylation of various xanthines and theophylline) [8]. The most popular plants and products containing caffeine are shown in Figure 1.

### 1.2. Metabolism of Caffeine

**Figure 2:**
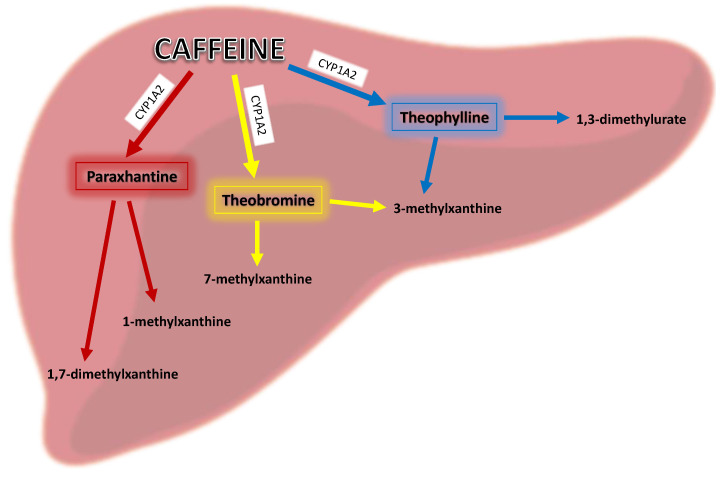
The main pathways of caffeine metabolism in the liver.

In the human body, caffeine is rapidly absorbed by the small intestine after oral administration into the body within 45 min and its average peak value occurs at 30 min [11], which directly depends on pH [8] and may be prolonged by food intake [12]. Its metabolic half-life is 3–5 h [13] and it readily penetrates the blood–brain barrier [12]. The first step in caffeine’s biotransformation is mediated by hepatic microsomal enzymes—selective catalysis by cytochrome P450PA in human liver microsomes [8]. Caffeine is primarily metabolized in the liver via the isoenzyme CYP1A2 (in about 80%), which causes its 3-demethylation to major metabolite, which is 1,7-dimethylxanthine (paraxanthine). Moreover, caffeine itself may increase CYP1A2 activity [14], and this isoenzyme is also responsible for the 1- and 7-demethylation of caffeine to 3,7-dimethylxanthine (theobromine) and 1,3-dimethylxanthine (theophylline), as shown in Figure 2. These metabolites may be further primarily demethylated via CYP1A2, then acetylated via N-acetyltransferase 2, and oxidized via xanthine oxidase or CYP3A4 to yield major metabolites that are excreted primarily in the urine including 1-methyluric acid, 5-acetylamino-6-formylamino-3-methyluracil, 1-methylxanthine (e.g., after further demethylation of paraxanthine via CYP1A2), 1,7-dimethyluric acid, and 1,7-dimethylxanthine (paraxanthine) [14]. A low percentage (0.5–4.0%) of an ingested dose of caffeine is excreted unchanged in urine and in bile and is also found in saliva, semen, and breast milk [8].

### 1.3. Genetics and Caffeine

Individual variation in response to caffeine consumption is connected with genetic aspects. There are two genes especially linked with caffeine metabolism—*CYP1A2* and *ADORA2A* [15]. The *CYP1A2* gene, which codes CYP1A2, is mainly responsible for caffeine metabolism, as above-mentioned. A single nucleotide polymorphism (SNP) (–163 C > A, rs762551) in intron 1 is considered responsible for individual differences in caffeine biotransformation [16]. Sachse et al. [16] determined that there is a homozygous variant A (AA)—“fast metabolizers”, a heterozygous variant (CA), and a homozygous variant C (CC)—“slow metabolizers”. Womack et al. [17] examined 35 male cyclists (16 AA homozygotes and 19 C allele carriers) and showed that there was a significantly greater performance improvement among men with AA genotypes. On the other hand, Pataky et al. [18] reported that athletes with the C allele had a better response to caffeine. Overall, most studies found that response to caffeine was not associated with CYP1A2—163 C > A polymorphism [19,20,21].

Gene *ADORA2A* encodes adenosine receptor A_2A_R, which plays a role in caffeine metabolism [22]. A 1976 T > C (rs5751876) SNP in the *ADORA2A* categorized people in TT—“high responders to caffeine” and CC/CT—“low responders to caffeine” [23]. Loy et al. [24] reported that TT athletes had higher improvements in cycling performance than C allele carriers. On the other hand, Carswell et al. [23] found that there were no differences in performance between TT and CT/CC genotypes. The results of the above investigations lead to the conclusion that further research into the influence of genetics on caffeine metabolism is needed. Future research should also be focused on detailed determination of which genes may affect the caffeine metabolism.

### 1.4. Effects on Receptors

Caffeine causes most of its biological effects via antagonizing all types of adenosine receptors (ARs): A_1_R, A_2A_R, A_2B_R, and A_3_R [25]. The blockade of adenosine receptors is observed in low concentrations of caffeine (<250 µM) [26]. Caffeine is also an agonist of ryanodine receptors (RyRs), stimulation of which increases the release of Ca^2+^ from the endoplasmic reticulum (ER) and a non-selective competitive inhibitor of phosphodiesterases (PDEs), the enzymes degrading cyclic adenosine monophosphate (cAMP), which leads to increases in cAMP concentration in the cell, but the effect of caffeine action with the stimulation of RyRs and the blockade of PDEs is possible only at higher doses (blood plasma concentration of 100 μM for RyRs and 2000 mg of caffeine intake for PDEs) [6,27]. Caffeine also interferes with γ-aminobutyric acid type A (GABAA) receptors [28], and may also exert anti-inflammatory activity by decreasing pro-inflammatory (CRP: C-reactive protein, interleukins (ILs): IL-1β, IL-6, IL-18, TNF-α: tumor necrosis factor α) and increasing anti-inflammatory (IL-10, adiponectin) marker levels [29,30].

### 1.5. Toxicity of Caffeine

A single dose consumption of 200 mg of caffeine, or less, by healthy people without comorbidities and pharmacokinetic disturbances, is usually not associated with toxic effects [31]. However, a dose above 300 mg at once can cause caffeine intoxication, the symptoms of which are mainly related to its stimulating effect. The most common ones are: restlessness, nervousness, excitement, insomnia, facial flushing, increased urination, gastrointestinal disorders, muscle tremors, chaotic flow of thoughts and speech, irritability, arrhythmia, tachycardia, and psychomotor agitation. The severity of the undesirable effects of caffeine consumption is dose dependent [32,33]. The threshold of caffeine toxicity appears to be about 400 mg/day in healthy adults (19 years or older), 100 mg/day in healthy adolescents (12–18 years old), and 2.5 mg/kg/day in healthy children (less than 12 years old) [34,35].

### 1.6. Adenosine

**Figure 3:**
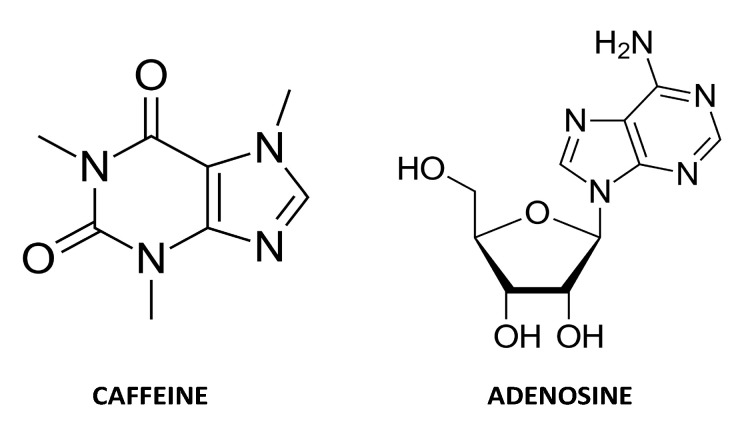
The comparison of caffeine and adenosine chemical structures.

Caffeine is so close in structure to adenosine that it is able to bind to the receptors that are specific to adenosine, which plays an important role in understanding how caffeine acts in the human body. Adenosine is an endogenous purine nucleoside, which is able to modulate the release of excitotoxic mediators, limit calcium influx, hyperpolarize neurons, and exert modulatory effects on glial cells when high concentrations of this nucleoside are observed [36]. Enhanced nerve activity, hypoxia, ischemia, or central nervous system damage may increase its level from 30–300 nM (physiological conditions) to 10 μM or even higher [37]. Adenosine binds to specific receptors expressed on the cell surface—A_1_R, A_2A_R, A_2B_R, A_3_R, which are members of G protein-coupled family receptors [38]. The A_1_ subtype is mainly localized in the brain, spinal cord, eye, adrenal gland, heart, and to a lesser extent in tissues such as skeletal muscle and adipose, while the A_2A_ subtype is mainly localized in the spleen, thymus, striatopallidal GABAergic neurons and to a lesser extent in the heart, lung, and blood vessels [39]. Caffeine causes most of its biological effects via antagonizing all types of ARs: A_1_, A_2A_, A_3_, and A_2B_ and, similar to adenosine, exerts effects on neurons and glial cells of all brain areas. As a consequence, caffeine, when acting as a nonselective AR antagonist, is doing the opposite of the activation of adenosine receptors due to the removal of endogenous adenosinergic tone [25,40,41]. The similarities between the caffeine and adenosine chemical structures are shown in Figure 3.

## 2. The Role of Caffeine in Various Systems in the Human Body

### 2.1. Central Nervous System

Caffeine has multiple targets in the brain such as adenosine, ryanodine, γ-aminobutyric acid receptors, and cyclic nucleotide phosphodiesterase isoenzymes. Its action on A_2A_Rs may explain the psychomotor stimulant effect, mediated by dopaminergic mechanisms. Caffeine, through antagonism of ARs, affects brain functions such as sleep, cognition, learning, and memory, and modifies brain dysfunctions and diseases: Alzheimer’s disease, Parkinson’s disease, Huntington’s disease, epilepsy, pain/migraine, depression, and schizophrenia [25]. Another possible mechanism of caffeine’s action on the nervous system is inhibition of the neurotransmitter acetylcholinesterase (AChE) [7]. This may be an important discovery for many studies on the effects of caffeine, clinically proven, but with unknown mechanisms of action. At low doses (<2 μg/mL in blood), caffeine stimulates the central nervous system but high blood concentration of caffeine (10–30 μg/mL) may produce restlessness, excitement, tremor, tinnitus, headache, and insomnia [8]. This conclusion was reported by Kaplan et al. [42], who observed that a lower dose (250 mg) of caffeine produced more positive effects (elation, peacefulness, pleasantness) than the higher dose (500 mg), which produced more negative effects (tension, nervousness, anxiety, excitement, irritability, nausea, palpitations, restlessness). Additionally, Watson et al. [43] documented the association between caffeine consumption and sleep quality in a group of 80 Australian adults (aged 38.9 ± 19.3 years). The study was based on a caffeine food frequency questionnaire and proved that higher doses of caffeine (192.1 ± 122.5 mg) were associated with worse sleep quality compared to lower doses of caffeine (25.2 ± 62.6 mg). 

#### 2.1.1. Caffeine Impact on Children and Adolescent

Caffeine intake has an impact not only on adults but also on children. In a subsequent study, Watson et al. [44] examined 309 Australian children (aged 8–12 years) on the basis of an interview conducted from the parents containing information about the consumption of caffeine (mostly in coffee, tea, and sodas) from 0 to 151 mg per day. This study showed that increased age, higher puberty scores, and higher morning tiredness values were associated with increased caffeine consumption. Otherwise, decreased total caffeine consumption was correlated with better internalization and decreased total behavior problems. Moreover, higher caffeine consumption was associated with a worse sleep routine, morning tiredness, and restless sleep. In another study, Richards and Smith [45] aimed to examine the connection between caffeine consumption and stress, anxiety, and depression in 3071 children from three academies in the southwest of England (aged 11–17 years). Participants were asked to complete a questionnaire about common dietary and caffeinated drink intake (total and separately) and well-being. Data analysis (made after adjusting additional dietary, demographic, lifestyle covariates, and sex) showed that caffeine consumption may be associated with stress, anxiety, and depression in secondary school children. Observed effects differed between men and women—effects relating to depression were strongest in females. Authors also reported that high caffeine intake (>1000 mg/week) was a risk factor associated with anxiety and depression in both sexes [45].

#### 2.1.2. Caffeine and Taste Perception

Caffeine can also affect taste perception. Circulating adenosine intensifies the sweet-tasting signals in the taste buds [46]. Caffeine blocks adenosine receptors [47] and negatively affects the perception of sweet tastes [48]. The bitter taste of caffeine may have an influence on how the human body reacts. The results of some studies suggest that bitter products can enhance performance and also give the signal to our brain that the organism is ready for action [49]. These aspects are still to be examined, and seem to be potentially important for research in the field of sports medicine. 

Gramling et al. [50] examined 12 males and 16 females (both caffeine consumers and non-consumers) using functional magnetic resonance imaging (fMRI), and showed differences in blood-oxygenation-level-dependent activation. The study was based on hedonic evaluation of caffeine, sucrose, or saccharin. During the hedonic evaluation of caffeine or sucrose, caffeine non-consumers had greater activation in neuronal areas associated with memory and reward, while during the hedonic evaluation of saccharin, greater activation in neuronal areas associated with memory, reward, and information processing occurred in the group of caffeine consumers. The results of the above studies support the observations on differential memory, reward, and information processing of taste between those who habitually consume caffeine and those who do not [50].

#### 2.1.3. Caffeine and Alzheimer’s Disease

Alzheimer’s disease is the reason for 50–70% of neurodegenerative dementia cases and is characterized by a progressive decline in cognitive function, and pathologically by loss of synaptic integrity and neurons, amyloid plaques composed of amyloid beta (Aβ), and neuronal tangles composed of hyperphosphorylated tau protein [47,51]. Li et al. [52] described a mechanism whereby caffeine protects against Aβ generation. This mechanism includes suppression of LDL cholesterol-enhanced amyloidogenic processing of amyloid beta protein precursor (AβPP) by blocking AβPP internalization via its actions on adenosine receptors—A_3_Rs. Additionally, neuronal cell cultures with Aβ in the presence of an A_2A_R antagonist completely prevented Aβ-induced neurotoxicity [53].

The neuromodulatory effects mediated by caffeine rely on a balanced activation of the inhibitory A_1_R and excitatory A_2A_R receptors [54]. There are also premises that caffeine may have a neuromodulatory effect via receptors RyRs and inhibition of PDEs, and this applies to all doses of caffeine within the range of normal, habitual consumption, so the only molecular targets for caffeine at nontoxic doses are the adenosine receptors in the brain, especially the inhibitory A_1_R and the faciliatory A_2A_R [55]. Upregulation of adenosine receptors A_1_R and A_2A_R is observed in AD. Activation of ARs affects synaptic neurotransmission and the release of various neurotransmitters (acetylcholine, glutamate) [53].

The consumption of some caffeinated drinks, and caffeine itself, is correlated with lower risk of AD and dementia. The neuroprotective effect of caffeine has been demonstrated by Maia et al. [56] in a study among 54 patients with AD who consumed 73.9 ± 97.9 mg/day of caffeine during the 20 years before diagnosis of AD in relation to individuals without AD who consumed 198.7 ± 135.7 mg/day during the corresponding 20 years of their lifetimes. Authors showed that caffeine consumption was associated with lower risk for AD. Kolahdouzan and Hamadeh [6] also stated that caffeine is protective in AD and the dosages of caffeine at which this effect is noticeable is 3–5 mg/kg body weight. According to Ritchie et al. [57], consumption of at least three cups of coffee per day is associated with less decline in verbal memory. Eskelinen et al. [58] also studied the association between caffeine intake and AD and dementia. A study among 1409 subjects, aged 50 years, showed that midlife coffee drinking reduced risk of dementia and AD by 62–70% in people who drank 3–5 cups of coffee per day, compared to low coffee consumers (0–2 cups). However, it was also shown that tea drinking is not associated with risk of dementia/AD. Other studies by Lindsay et al. [59] in the Canadian community of 10,236 participants over the age of 65 years stated that coffee consumption was correlated with reduced risk (31%) of AD in the Canadian population. Overall, it should be emphasized that active constituents of coffee or tea, other than caffeine, may contribute to their effects on cognition/AD risk [59]. Recently, Dong et al. [60] investigated the association of intake of coffee (caffeinated and decaffeinated) and caffeine derived from caffeinated coffee with cognitive performance in older adults. They analyzed data from the National Health and Nutrition Examination Survey (NHANES) 2011–2014 that included a total of 2513 participants aged 60 years or older. The information on coffee and caffeine consumption were received from two 24-h dietary recalls, whereas cognitive performance was evaluated by the Consortium to Establish a Registry for Alzheimer’s Disease (CERAD) test, Animal Fluency test and Digit Symbol Substitution Test (DSST). The authors observed no significant association between decaffeinated coffee intake and various dimensions of cognitive performance. It was suggested that caffeinated coffee and caffeine from coffee were associated with cognitive performance, while decaffeinated coffee was not associated with them [60]. Confirmation of the above findings was a placebo-controlled study involving sixty elderly people that showed that there was no significant association between decaffeinated coffee and cognitive functions [61]. In contrast, in a randomized, placebo-controlled study provided by Haskell-Ramsay et al. [62], it was shown that decaffeinated coffee may have a protective effect on cognitive performance. It should be noted, however, that the final analysis was carried out on a relatively small number of male and female participants: older (61–80 years, *N* = 30) and young (20–34 years, *N* = 29).

The results of studies on the effect of caffeine on the nervous system and cognitive functions are very promising, thus it would be justified to include this substance in the algorithm of management in people exposed to neurodegenerative diseases. However, taking into account the above data, a detailed study on the effects of caffeine and caffeine-containing products must first be carried out, as these products can contain many various bioactive ingredients that can alter the primary effect of caffeine.

#### 2.1.4. Caffeine and Parkinson’s Disease

Parkinson’s disease (PD) is a neurodegenerative disease with motor and non-motor symptoms [63]. Some studies reported that caffeine intake is beneficial for PD patients, similarly to Alzheimer’s disease. First of all, caffeine intake is associated with lower risk of Parkinson’s disease. Ross et al. [64] analyzed data on 8004 Japanese-American men (aged 45–68 years) drinking at least 28 ounces (421 mg of caffeine) of coffee, who were 5-times less exposed to PD, and this risk decreased as coffee consumption increased. Another study by Liu et al. [65] examining 187,499 men and 130,761 women showed that higher caffeinated coffee consumption was associated with lower PD risk in a dose-dependent manner, but consumption of other caffeine-containing beverages (soft drinks, hot tea, and iced tea) was not associated with the risk of PD. Moreover, Palacios et al. [66], after adjustment for age, smoking, and alcohol intake showed that the association of caffeine consumption with lower risk of PD was stronger for men compared to woman. These findings may be explained by the combined effects of caffeine and other coffee substances and should be further investigated. Although caffeine may reduce the risk of Parkinson’s disease, its intake does not improve the motor symptoms of the disease, as postulated by Postuma et al. [67], who treated 60 PD patients with caffeine and 61 with placebo. Patients with PD ongoing for 1–8 years consumed 200 mg of caffeine twice a day through 6–18 months. The authors found that caffeine does not influence sustained motor improvement in PD [67]. Caffeine not only reduces risk of PD, but may also be a promising diagnostic biomarker for early PD. Fujimaki et al. [63] examined 108 patients with PD and 31 age-matched healthy controls by using liquid chromatography-mass spectrometry to measure levels of caffeine and its 11 metabolites in serum. The authors showed that the absolute serum concentrations of caffeine and nine of its downstream metabolites were significantly lower in patients with PD than in healthy controls [63]. The above observations show new possibilities for laboratory diagnostics, but more research is needed for confirmation of those observations and complete evaluation of these findings.

There are also indications that caffeine may be an adjuvant in the treatment of PD. It was hypothesized that caffeine may increase excitatory activity in local areas by inhibiting astrocytic inflammatory processes, but evidence remains inconclusive. Roshan et al. [68] suggested that the co-administration of caffeine with currently available PD drugs helps to reduce drug tolerance and increases the effectiveness of the drug action. Despite the positive aspects, caffeine in combination with other substances can sometimes have a negative effect. Simon et al. [69] examined 1549 patients with early PD who completed a caffeine intake questionnaire. Half of the examined subjects were treated with creatine (10 g per day). Studies showed that higher caffeine intake was associated with significantly faster PD progression among subjects taking creatine [69], most probably caused by the caffeine action, which completely negates the effect of creatine on muscle contractions [70,71], potentially by counteracting the creatine-associated facilitation of calcium uptake by the sarcoplasmic reticulum [71].

#### 2.1.5. Caffeine and Huntington’s Disease

Huntington’s disease (HD), an inherited neurodegenerative disorder caused by expanded CAG (cytosine, adenine, guanine) repeats, is characterized by motor, cognitive, and psychiatric disturbances [72]. HD is caused by a mutation in the *IT15* gene that encodes for the protein huntingtin (Htt), which is widely distributed in the central nervous system, but to date, the exact cellular function of Htt is still not completely understood [73,74]. Htt is involved in many physiological functions including brain-derived neurotrophic factor expression/transport. When the Htt protein becomes mutated (mHtt), not only is normal Htt function impaired, but several mechanisms important for neuronal activity and survival become impaired, leading to a gain of function that can be toxic to the cells [75]. It is not clear whether HD is a prion-like disorder comparable to Alzheimer’s or Parkinson’s diseases, but experimental data suggests that mHtt triggers mis-conformation of wild-type Htt, and neuropathological observations in patients who received intracerebral allografts support the transfer of HD pathology from cell to cell [76]. 

In their review, Blum et al. [74] showed that many genetic, epidemiological, and experimental studies suggest that adenosine receptors, both A_1_R and A_2A_R, are linked to HD pathophysiology, although their exact involvement remains unclear. Additional investigations are needed to dissect the pre- and postsynaptic aspects of A_2A_R, and the relationship between A_2A_R and mHtt induced glial dysfunction, which has been largely underestimated. Since HD is a chronically progressive disease, there are multiple mechanisms along the degenerative process that may be affected by their interactions with A_2A_R. The role of A_1_R in HD pathogenesis also needs to be reconsidered. 

Simonin et al. [72] assessed caffeine consumption in 80 patients with HD and it turned out that among people who consumed a caffeine dose greater than 190 mg per day, the risk of earlier HD was higher. As ARs, especially A_1_R and A_2A_R, are known targets for caffeine, the possible mechanism of caffeine action in HD pathophysiology may be associated with its activity as a nonselective AR antagonist. Chronic caffeine intake may reduce adenosine A_2A_R activity [77]. In the 3-NP model of Huntington’s disease, A_2A_R antagonism has been associated with worsening signs [78]. However, Tanner et al. [79], based on their study results, concluded that only caffeinated soda, but not other caffeinated beverages, was associated with Huntington’s disease risk, nor was a combined caffeine dose associated, but this finding may be spurious, or not related to caffeine.

#### 2.1.6. Caffeine and Perception of Pain

Caffeine is a constituent of many over-the-counter pain relievers and prescription drugs because the vasoconstricting and anti-inflammatory effects of caffeine act as a compliment to analgesics, in some cases, increasing the effectiveness of pain relievers by up to 40% [80]. Studies conducted on 62 people (aged 19–77 years) by Overstreet et al. [81] showed that habitual dietary caffeine consumption was associated with a higher pain threshold, higher heat pain tolerance, and higher pressure pain threshold. The mechanism by which caffeine can reduce pain sensation appears to be closely related to its direct effects on adenosine receptors, especially through the central blocking of those receptors that influence pain signaling or block peripheral adenosine receptors on sensory afferents. The antagonism of adenosine receptors as well as the inhibition of cyclooxygenase activity in some places may explain the anti-nociceptive effect of caffeine and its supportive effect [82,83,84,85,86,87]. This is the reason why low-dose caffeine is present as an adjuvant in conjunction with antidepressants, acetaminophen, and non-steroidal anti-inflammatory drugs in many over-the-counter (OTC) pain medications [88,89].

#### 2.1.7. Caffeine and Mental Health 

Depression, anxiety, and suicide are becoming an increasingly common problem among children and adults. This prompts not only the search for new therapies, but also for the causes of these ailments more closely. Over the years, caffeine has been investigated as a potential protective or risk factor for psychiatric disorders [90]. It has already been documented that caffeine intake is associated with depressive symptoms. Lucas et al. [91] indicated that the risk of depression is associated with the dose of consumed caffeine. They showed that people who drank more than two cups of caffeinated coffee per day were 24% less exposed to depression than those who did not drink coffee. In addition, Richards and Smith [45] found that the incidence of depression decreased as the dose of caffeine intake increased, but they did not verify the differences between men and women. Botella et al. [92] investigated the effect of caffeine on depression in 2307 students aged 11–17 and showed that consuming less than 1000 mg of caffeine per week increased the risk of depression in girls, but not in boys. This conclusion has been confirmed by Iranpour and Sabour [93], who analyzed data derived from 4737 adults and found that women who consumed more caffeine had a lower risk of depression. 

Kendler et al. [94] analyzed data from the Virginia Twin Registry of about 3600 adult twins and reported that the risk of developing anxiety increased after consumption of >6 cups of caffeinated coffee per day. Additionally, Bertasi et al. [95], in their study among 114 college students, showed that high caffeine intake is associated with higher levels of anxiety. Botella et al. [4] examined 3323 students (11–17 years old) and showed that the effect of caffeine on anxiety is significant mainly in boys—the anxiety increased with increased dose of caffeine intake. According to this, Hedstrom et al. [96] obtained similar results among 39 men and 60 women aged 18–31 years, and reported that at the same doses of caffeine consumption, men had a higher anxiety state than women. 

Some studies have examined the effect of a single instance of caffeine intake on neurocognitive performance. Konishi et al. [97] investigated the effect on driving performance in 100 healthy Japanese volunteers (50 males, 50 females, aged 22–59 years) with a valid driver’s license. In this double-blinded, randomized, placebo-controlled study, half of the participants formed a caffeine intake group and the other a placebo group. Individuals did not consume caffeine for three days before the examination, then the “caffeine group” was given 200 mg of caffeine. Thirty minutes after administration, cognitive functions were evaluated via the Symbol Digit Coding Test (SDC), the Stroop Test (ST), the Shifting Attention Test (SAT), and the Four Part Continuous Performance Test (FPCPT). After these tests, the authors checked driving performance (brake reaction time and standard deviation of the lateral position) using a driving simulator. Research showed that the “caffeine group” had more appropriate responses than the placebo group on the SAT, made less mistakes, and had shorter times in the brake reaction time test. Studies conducted by Yu et al. [98] using long-term self-renewing neuroepithelial stem cells showed that consumption of caffeine (3 μM and 10 μM) activates immediate early genes after 1 h, while neuronal projection development processes were upregulated and negative regulation of axon extension processes were downregulated at 3 h.

It is known that caffeinated coffee consumption may be associated with a lower risk of suicide, depending on the amount of coffee consumed daily [99,100]. Lucas et al. [91] analyzed three prospective cohorts of American adults consisting of 43,599 men and 164,825 women, among which caffeine consumption was tested every four years. The authors documented 277 deaths from suicide and showed correlations between caffeine intake and deaths as an inverse relationship [91]. However, in a Finnish population study [101] of over 43,000 people who were followed for an average of 14.6 years, a J-shaped relation was found between daily coffee drinking and the risk of suicide. Compared to those who drank one cup of coffee per day, the risk of suicide was lower with moderate coffee consumption (2–3 cups per day to 6–7 cups per day), but increased with higher consumption (8–9 and 10 cups per day). The main effects and probable mechanisms of caffeine action on the nervous system are shown in Table 1.

In conclusion, caffeine action in a variety of central nervous system diseases and disturbances is multi-directive, as caffeine has multiple targets in the brain and affects many brain functions such as sleep, cognition, learning, and memory, while on the other hand modifying brain dysfunctions and diseases such as Alzheimer’s disease, Parkinson’s disease, Huntington’s disease, epilepsy, pain, and depression. The effect of caffeine depends mainly on the amount of the substance. At low concentrations, it has a positive effect on the human brain, but higher doses may be responsible for negative effects in mood and behavior. In some diseases, caffeine consumption in high doses may be a risk factor (AD, PD, Huntington’s disease), however, on the other hand, thanks to its therapeutic properties and ability to antagonize ARs, caffeine may be useful in the treatment of PD and pain symptoms or, in connection with some other medications, may delay the progression of the disease. The utility of caffeine and its metabolites in laboratory diagnostics as markers of certain diseases (e.g., PD) also seems promising.

### 2.2. The Immune System

The immune system is complex and is made up of various cells and proteins that are responsible for the body’s resistance, particularly through the immune response, which is a weapon against foreign antigens [103]. More and more attention is paid to the two-way relationship between the immune system and diet [104]. The role of caffeine in the functioning of the immune system reaches the cells themselves. Gibbs et al. [105] reported that caffeine in high concentrations (5 mM) inhibits the mammalian target of rapamycin (mTOR) in human myeloid leukemia cells, primary human acute myeloid leukemia cells, and primary human basophils, and affects glycolysis and the release of pro-inflammatory cytokines. Additionally, in monocytes, the effect of caffeine was potentiated by its ability to inhibit xanthine oxidase (purine catabolism), and in basophiles, caffeine increased intercellular cAMP levels [105].

Adenosine receptors are expressed on monocytes and macrophages and through these receptors, ligands (e.g., adenosine, caffeine) can modulate monocyte and macrophage function [38]. Chavez-Valdez et al. [106] used cord blood monocytes (CBM) from 28 full-term infants (>37 weeks’ gestation at birth) to investigate changes in the ARs mRNA profile (by qRT-PCR) and protein expression (by western blot) after in vitro culture, caffeine, or lipopolysaccharide (LPS) exposure, and the modulation of cytokine release and cAMP production (by ELISA) induced by caffeine and ARs antagonists. After 48 h in culture, it turned out that caffeine (50 μM) decreased tumor necrosis factor-alpha (TNF-α) from LPS activated-CBM by 20% and *TNF-α* gene expression by 30%, in conjunction with a minimum 2-fold increase in cAMP. This suggests that caffeine increases cAMP production and inhibits pre-transcriptional TNF-α production by CBM via A_1_R blockade. These observations were later confirmed by Chavez-Valdez et al. [107], where the authors used LPS-activated CBM from 19 infants and exposed them to caffeine in various concentrations (0–200 μM) with or without previous exposure to ARs antagonists, and showed that caffeine at ≤100 μM reduced TNF-α levels by 25% and IL-10 levels by 17–25%. The results of the same study confirmed that caffeine concentration is directly positively correlated to toll-like receptor 4 (TLR4) gene expression, and therefore it promotes inflammation [107]. Steck et al. [108] demonstrated that caffeine may act as a phosphodiesterase inhibitor, suppressing phagocytosis in mononuclear phagocytes by promoting an anti-inflammatory response. Although the obtained results were promising, in the human body, it is a complex and dynamic process that involves many pathways that may contradict each other, resulting in inconsistent results dependent on dosage of caffeine and state of the exposed cell. In their study, Shushtari et al. [109] used mesenchymal stem cells (MSCs) isolated from bone marrow and treated them with different concentrations of caffeine (0, 0.1, 0.5, and 1 mM) for 72 h, out of which 24 h consisted of incubation with macrophages. The authors observed that MSCs treated with caffeine enhanced phagocytosis and the expression of reactive oxygen species, nitric oxide, and IL-12 by macrophages. These results proved that caffeine augments the instruction of anti-inflammatory macrophages and showed the potential mechanism of the immunomodulatory and anti-inflammatory effects of caffeine [109]. However, the presented results indicate the therapeutic properties of caffeine, high doses of caffeine necessary to obtain certain effects, may be toxic to the human body.

In summary, caffeine reaches cells by interaction with ARs, which are expressed on them. Its anti-inflammatory effect is important in the functioning of the immune system, however, more attention is required to the potential use of caffeine as a therapeutic agent in hematological diseases related to the malfunction of the immune system.

### 2.3. Digestive System

**Figure 4:**
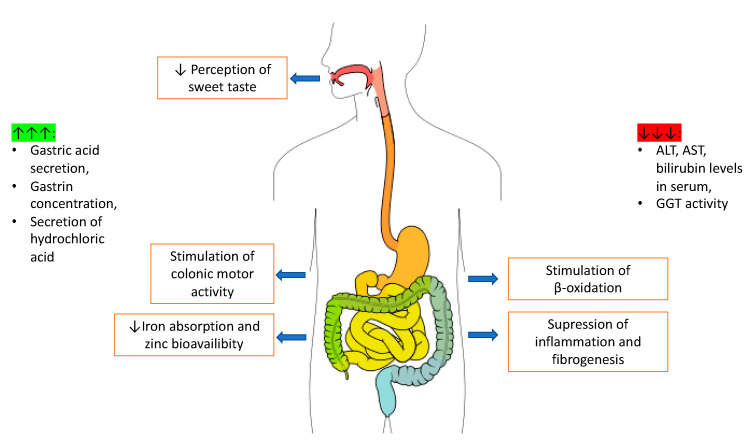
The scheme of the main effects of caffeine action on the digestive system. ALT—alanine aminotransferase, AST—aspartate aminotransferase, GGT—γ-glutamyltransferase. ↓↓↓—decrease, ↑↑↑—increase.

Caffeinated coffee consumption is one of the causes of gastrointestinal discomfort reported by patients as well as digestive system problems noted by doctors. The main pharmacologically active substance in coffee is caffeine, which may increase gastric acid secretion [8], relax smooth muscles by increasing gastrin concentration [110], and stimulate the secretion of hydrochloric acid [111], causing higher risk of inflammation of the intestinal mucosa and stomach. On the other hand, caffeine is said to be antioxidant and to have anti-inflammatory activity [112], thanks to which caffeine can reduce alanine aminotransferase (ALT), aspartate aminotransferase (AST), and bilirubin level in serum [112]. There is also a connection between coffee consumption and γ-glutamyltransferase (GGT) activity—together with higher coffee consumption, lower GGT activity was observed [113]. It should be noted, however, that coffee ingredients may disturb iron absorption [114], and zinc’s bioavailability [115]. It is suggested that coffee and its compounds may also affect intestinal microbiota, especially *Bacteroides*, and increase its level [116]. The effects of caffeine action on the digestive system are schematically shown in Figure 4.

#### 2.3.1. Caffeine Action on the Small and Large Intestine

Caffeine has an impact on net fluid movement and transit times, although the data in this respect are not conclusive. It has been reported that caffeine ingestions (75–300 mg) caused increased net secretion in jejunum for a minimum of 15 min, and 35 min later in ileum in the same doses of caffeine [117]. The results of another study showed that caffeine affects esophageal function by decreasing the pressure on the lower esophageal sphincter, leading to its relaxation [118]. Relaxed lower esophageal sphincter may be a reason for gastric reflux [119]. Moreover, it was also documented that caffeinated coffee stimulates gallbladder contraction and colonic motor activity, but there were no connections between coffee consumption and dyspepsia [119].

It is still unclear whether and to what extent the consumption of caffeinated coffee and caffeinated products affects gastrointestinal transit time and whether this is an effect of caffeine content. Boekema et al. [119] investigated the effect of caffeinated coffee consumption on gastrointestinal motility, on gastric emptying, and oro-cecal transit time. In a randomized, controlled, cross-over study gastric emptying and oro-cecal transit time, the authors studied 12 healthy volunteers using applied potential tomography and the lactulose hydrogen breath test. The lag-phase duration after coffee intake was not significantly different from that after water (median 19.8 min vs. 19.3 min, respectively), nor was the gastric half-emptying time (median 75.7 min vs. 83.4 min, respectively). Furthermore, coffee had no significant effect on oro-cecal transit time (median 135 min vs. 140 min, respectively). No significant correlation between any of the examined parameters and mean daily caffeinated coffee intake was found, which confirms that coffee consumption does not affect gastric emptying of a liquid meal or small bowel transit. On the other hand, Rao et al. [120], who investigated the effects of caffeinated coffee on colonic motor activity in healthy humans, revealed that coffee stimulates colonic motor activity. Its magnitude was similar to a meal, 60% stronger than water, and 23% stronger than decaffeinated coffee.

#### 2.3.2. Caffeine and the Liver

Animal models (mice and rats) have shown that caffeine may play a potential role in the stimulation of β-oxidation in hepatic cells, intrahepatic lipid content reduction, and hepatic autophagy [5,6], which suggests that caffeine ingestion suppresses inflammation and lipogenesis [121], reduces lipid peroxidation [112], and is linked with lower risk for nonalcoholic fatty liver disease [122]. Caffeine is inversely associated with the risk of cirrhosis [123] and the risk of hepatocellular carcinoma, especially among people with the hepatitis C virus and other liver diseases [124]. Modi et al. [125] examined 177 patients (among which 99 were male, 104 Caucasian, 121 with hepatitis C) who underwent liver biopsy and completed the caffeine questionnaire. The authors analyzed caffeine consumption by coffee-cup equivalents, and revealed an association between caffeine consumption and lower risk of liver fibrosis, especially in patients with HCV infection when two coffee-cup equivalents were consumed per day [125]. Li et al. [126] examined the molecular aspects of the protective role of caffeine in fibrogenesis by using a LX-2 cell line (immortalized human hepatic stellate cells) and exposing it to various concentrations of caffeine. The results of the study showed that caffeine inhibits the viability and increases the apoptosis of the LX-2 cells in a dose- and time-dependent manner by autophagy of endoplasmic reticulum, via the inositol-requiring enzyme 1α signaling pathway. In another study, Salomone et al. [117] suggested that through stimulation of the nuclear factor erythroid 2-related factor 2 (Nrf2) (which reduces oxidative stress and inflammation), peroxisome proliferator-activated receptors alfa (PPARα) (which increase β-oxidation and reduce lipid accumulation), 5’ adenosine monophosphate-activated protein kinase (AMPK) (which increases autophagy and reduces lipid accumulation), and inhibition of sterol regulatory element-binding transcription factor (SREBP1c) (which reduces *de novo* lipogenesis and reduces lipid accumulation) and receptor A_2A_R, coffee and its components suppress liver fibrogenesis and carcinogenesis [117]. Moreover, caffeine, depending on the dose, is said to increase apolipoprotein A1 and paraoxonase-1 protein levels in liver cells [127].

#### 2.3.3. Caffeine and Glycaemia

High doses of caffeine increase glucose tolerance [128] and decrease insulin sensitivity [129]. It has been shown that decaffeinated coffee, in contrast to caffeinated, reduces HbA_1c_ levels [130] used in glycaemia monitoring, which suggests that coffee contains other substances that may improve glucose metabolism, and this needs to be examined. The studies on caffeine consumption among people with type 2 diabetes do not show any correlation between decaffeinated coffee and improvement in glycemic control [130], and should be confirmed by additional research, since the results of another study have shown that frequent (at least seven cups per day) coffee consumption may reduce the risk of type 2 diabetes by 50% [131], as confirmed in many studies [132,133,134]. It has also been documented that caffeine may affect the phosphatase activity of glycogen synthase [110]. When linked with glucose, there was only slight stimulation over a wide concentration range, thus it seems that there is no synergy between these two compounds in the activation of glycogen synthase phosphatase [135].

#### 2.3.4. Caffeine and Digestive Tract Cancer

Some studies indicate that coffee with caffeine may reduce the probability of digestive tract cancer occurrence, especially liver cancer [117,136] and colon cancer [137]. Liu et al. [114] used human gastric cancer cells in in vitro studies to show that caffeine treatment suppressed gastric cancer cell growth and viability, and activated the caspade-9/-3 pathway, which induced apoptosis. Taking the above into consideration, it seems that caffeine may be useful as a sustained anticancer agent in the therapy of gastric cancer, however, these observations should be confirmed by additional studies. The meta-analysis conducted by Li et al. [138] consisting of 25 case-control and 16 cohort studies showed that the risk of colorectal cancer was reduced by 15% for the highest coffee drinkers compared to low or non-drinkers, and the authors concluded that the risk of colon cancer was reduced by 21%. In this meta-analysis, information on the classification of subjects to the group of high, low, or non-coffee drinkers was taken by the authors from the classifications described previously in other, original articles, and thus there was no possibility of assigning unambiguous values for these groups. Tian et al. [139] reported a significant decrease in the risk of colorectal and colon cancer among subjects consuming at least four cups of coffee a day. The results of meta-analysis conducted by Sang et al. [140] showed that the risk of liver cancer for high coffee drinkers was 50% lower than for non/almost never drinkers. Additionally, Bravi et al. [141] analyzed some studies and in their meta-analysis showed that the risk of hepatocellular carcinoma was reduced by 40% for any coffee consumption versus no consumption. The authors suggested that the effect on liver enzymes and development of cirrhosis may be responsible for the protective effect of caffeine against liver carcinogenesis [141].

As documented by the many studies above-mentioned, caffeine action covers a variety of points in the food pathway, starting from the mouth, through the stomach, small intestine, and liver to the large intestine. It increases the secretion of gastric acid, decreases the activity of liver enzymes, and improves the motor functions of the intestines. Caffeine’s antioxidant and anti-inflammatory properties reduce the risk of liver disease (cirrhosis, fibrogenesis, nonalcoholic fatty liver disease, etc.) and some cancers. Additionally, caffeine is considered to be a promising anticancer agent because of its antioxidant potential, which is especially important in cancer treatment because cancer progression and metastasis are accompanied by oxidative–antioxidant imbalance.

### 2.4. Respiratory System

The effect of caffeine on the respiratory system is mainly described as beneficial, regardless of whether it is administered individually, as a component of drugs, or synergistically with applied therapy [142]. Caffeine acute ingestion (3 mg/kg body weight) may improve peak aerobic performance and increase peak pulmonary ventilation. The role of caffeine in this effect may be explained by its ability to affect respiratory muscle [143]. Supinski et al. [144] examined the effect in six healthy people of single, orally administered doses of caffeine (600 mg) on diaphragmatic muscle, and showed that caffeine increased trans-diaphragmatic pressure, probably by increases in muscle contractility. This suggests that caffeine may be used in the treatment of patients with respiratory muscle weakness. Another possible mechanism of caffeine action is without a doubt its influence on receptors. For example, Bruce et al. [145] showed that caffeine (200 mg) caused a decrease in exhaled nitric oxide via AR antagonism, or by altering levels of cGMP. The main effects of caffeine action in respiratory system diseases are summarized in Table 2.

#### 2.4.1. Caffeine and Asthma and Chronic Obstructive Pulmonary Disease

An important action of caffeine is the stimulation of the respiratory system, hence caffeine is a common ingredient in bronchodilators [146]. Welsh et al. [146] examined 75 people with mild to moderate asthma and showed that caffeine (even at less than 5 mg/kg body weight) improved lung function for up to two hours after consumption (differences in forced expiratory volume in one second about 5%). The authors concluded that caffeine improves airway function modestly, for up to four hours, in people with asthma [146].

There is little information about the association of caffeine with chronic obstructive pulmonary disease (COPD). Hirayama et al. [147] examined 277 Japanese COPD patients (aged 50–75 years) who drank more caffeinated coffee and had a significantly higher mean caffeine intake (311.3 ± 176.2 mg/day) than the control group (278.4 ± 188.1 mg/day). Relative to the control group of non-drinkers (340 individuals), the risk of COPD apparently increased for those drinking at least two cups of coffee daily. Similarly, total caffeine intake was associated with the prevalence of COPD—for consuming over 312 mg/day, the risk of COPD was higher when compared to a low intake of less than 184 mg/day. In their retrospective study, Lopes et al. [148] evaluated the effect of chronic caffeine consumption on the risk for COPD exacerbations among 90 patients with COPD and showed that mean caffeine consumption (149.7 ± 140.9 mg/day) was not associated with an effect on the frequency of COPD exacerbations. Due to the small amount of research on the effects of caffeine on COPD, the results of the above studies should be confirmed in further investigations.

#### 2.4.2. Caffeine and Breathing Problems

Aranda et al. [149] in their study examined 12 infants with infantile apnea and observed significant increases in ventilation, tidal volume, and mean inspiratory flow with plasma concentrations of caffeine ranging from 8 to 20 mg/L. The above results show that caffeine may be valuable medicine, but more studies are required for confirmation of these findings. Another study was conducted by Kassim et al. [150] among 18 prematurely born infants being weaned from mechanical ventilation. The infants were given caffeine (5 mg/kg body weight/24 h), and after 6 h, measurements were made. The maximum pressures generated by occlusions at end inspirations and end expirations, and lung volume, had significantly improved. This suggests that caffeine administration increases respiratory muscle function, and is associated with lung function improvement. Davis et al. [151] measured the effect of caffeine upon pulmonary mechanics in 16 infants with bronchopulmonary dysplasia. A dose of 10 mg/kg body weight of caffeine caused a 37% increase in minute ventilation, 42% increase in tidal volume, and 47% improvement in total pulmonary compliance. Total lung resistance decreased by 20% [151].

#### 2.4.3. Caffeine and Lung Cancer

Baker et al. [152] examined 993 individuals (624 male and 369 female) with primary, incident lung cancer and observed an elevated lung cancer risk for drinkers of at least two cups of caffeinated coffee per day, as confirmed in a meta-analysis conducted by Wang et al. [153]. The authors reported a linear relationship between coffee consumption and increased risk of lung cancer, especially for consumers of ≥3 cups coffee per day [153].

In summary, the importance of caffeine in the functioning of the respiratory system is great due to the important function this system plays not only for basic life functions, but also in the world of sports, where it seems important to consider caffeine as a means of increasing ventilation, tidal volume, and airway functions. Thanks to these effects, caffeine can be also used as a medication for people with asthma, but it should be considered that caffeine can also have negative effects on the respiratory system such as increasing the risk of COPD and lung cancer. Therefore, once again, we can see that although caffeine has many benefits, it is necessary to exercise moderation in consuming products that contain it.

### 2.5. Circulatory System

In general, an acute intake of caffeine stimulates a modest increase in blood pressure (both systolic and diastolic) [154]. In volunteers who abstained from caffeine-containing products, a bolus dose of 250 mg led to a 5–10% increase in both systolic and diastolic blood pressure for 1–3 h. Tolerance to this effect developed, however, when caffeine was given three times a day for seven days [155,156]. In another study, Van Dusseldorp et al. [157] reported that daily use of decaffeinated coffee (40 mg caffeine) instead of five cups of regular coffee (445 mg caffeine) for six weeks led to a small but significant decrease in systolic (by 1.5 mmHg) and diastolic (by 1.0 mmHg) blood pressure in 45 healthy volunteers. Moreover, scientists from Radboud University Nijmegen Medical Center observed that the dose of caffeine in coffee alone raised blood pressure less than an identical concentration given in the pill [158]. These effects suggest that the mechanism of caffeine action is an increase in intracellular calcium concentrations, the release of norepinephrine, and the sensitization of dopamine receptors, because caffeine has a positive inotropic effect [159]. Some studies have documented that coffee consumption and the type of coffee plays a role in lipid metabolism. Boiled coffee increases serum total and LDL cholesterol concentrations, but filtered coffee does not significantly change serum cholesterol levels [160]. A study of 32 healthy volunteers of both sexes (aged 25.2 ± 4.2) with body mass index (BMI = body mass (kg)/height^2^ (m)) of 21.7 ± 2.2, conducted by Melik et al. [161], showed that 200 mg of caffeine consumption increased arterial pressure and decreased heart rate and resting cutaneous laser-Doppler flux. It was observed that myogenic activity also increased. In conclusion, the results of this study suggest that caffeine affects cutaneous microvascular function during rest and during a post-occlusive reactive hyperemia response [161].

#### Caffeine and Arrhythmia

One of the possible mechanisms of caffeine action is blocking ARs (mainly subtypes A_1_ and A_2_) [47] and causing a higher release of dopamine and noradrenalin [162]. It inhibits the action of natural adenosine, and can cause tachycardia and arrhythmias due to the increased activation of the β1-receptor [163]. As a nonselective competitive inhibitor of A_2A_Rs, caffeine might attenuate the vasodilator effect of adenosine, and increase sympathetic activity, yielding to capillary de-recruitment and leading to decreased myocardial perfusion reserve [47,164]. Seitz et al. [165] in their study showed that caffeine reduced the myocardial perfusion reserve index (MPRI). The authors examined 25 patients (84% male, median age 69 years) with substantial myocardial ischemia and coffee addiction (3–4 cups per day), who underwent repeat adenosine stress perfusion imaging by cardiovascular magnetic resonance after a prior intake of two cups of coffee (about 200 mg of caffeine) 1 h before examination, which confirmed the need for abstinence from caffeine [165].

Caffeine is also a nonspecific inhibitor of phosphodiesterases that is able to intensify production of cAMP and cGMP, which affects cardiac contractility, and this may predispose to arrhythmias [163]. The meta-analysis conducted by Greenland [166] was based on 22 studies, and the author showed that drinking at least five cups of coffee per day may enhance the risk of myocardial infraction or coronary death.

In conclusion, the circulatory system is one of the most vulnerable to the negative effects of caffeine because of its effects on blood pressure (both systolic and diastolic), which can be rapidly increased. It should be emphasized that the type of coffee (caffeinated or decaffeinated) and the way that caffeine is served influence blood pressure, and it was suggested that the stimulating effect of caffeine action is associated with an increase in intracellular calcium concentrations, the release of norepinephrine, and the sensitization of dopamine receptors. Caffeine is able to inhibit ARs and PDEs and activate the β1-receptor, which can lead to problems related to cardiac function such as tachycardia and arrhythmia and, consequently, to death.

### 2.6. Urinary Tract

**Figure 5:**
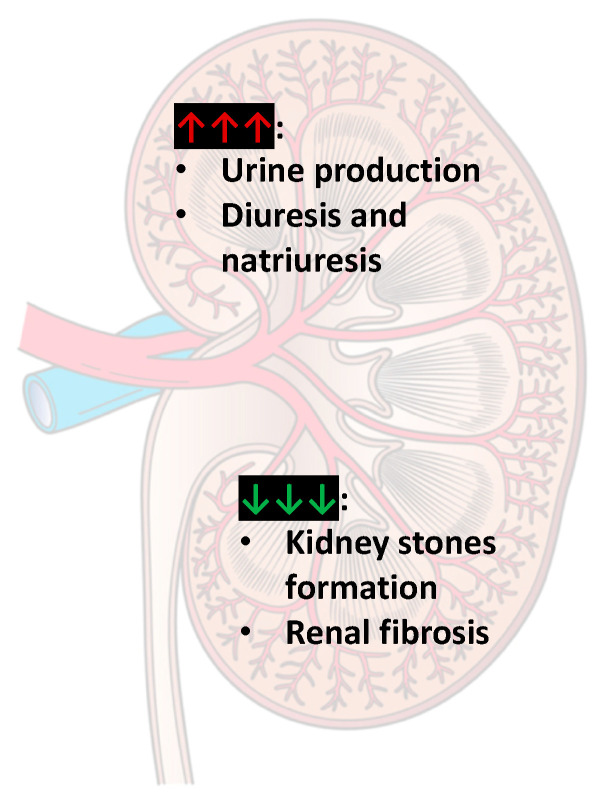
The main effects of caffeine action on the kidneys. ↓↓↓—decrease, ↑↑↑—increase.

The well-known diuretic effect of caffeine is related to the maintenance of the water–salt balance in different segments of the nephron in which adenosine plays a complex role, depending on the differential expression of its receptors. Therefore, caffeine increases the rate of glomerular filtration, counteracting by the vasoconstriction of renal afferent arteriole mediated by adenosine via type 1 AR during tubuloglomerular feedback. It should also be emphasized that caffeine inhibits Na^+^ reabsorption at the level of the proximal renal tubules and disrupts the hepatorenal reflex through sensory nerves in Mall’s intrahepatic spaces [167]. Caffeine may increase urine production [8], and excessive caffeine intake (more than 400 mg/day) may increase the risk of detrusor instability (unstable bladder) in women [168]. In the kidneys, caffeine induces diuresis and natriuresis [169]. Wu et al. [170] showed that there was a positive correlation between several urinary caffeine metabolites, especially paraxanthine, theobromine, and caffeine and urine flow rate. Moreover, the number of metabolites showing certain flow-dependency was higher in males than females and was higher in young participants compared to elderly participants [170]. A study by Lohsiriwat et al. [171] on nine women and three men (aged 21–40 years) with overactive bladder symptoms who drank 8 mL/kg body weight of water with or without caffeine at two separate sessions showed that caffeine at 4.5 mg/kg body weight caused diuresis and decreased the threshold of sensation at the filling phase, with an increase in flow rate and voided volume. In conclusion, caffeine can promote early urgency and frequency of urination [171]. The main effects of caffeine on kidney action are shown in Figure 5.

#### 2.6.1. Caffeine and Urinary Incontinence

Jura et al. [172] conducted a prospective cohort study of 65,176 women (37–39 years old) without urinary incontinence, using questionnaires to measure caffeine intake. The authors associated risk for urgency incontinence with high caffeine intake and showed that this risk was higher in patients who consumed more than 450 mg of caffeine, and 25% of incident urgency incontinence may be attributable to caffeine consumption. One year later, in 2012, Hirayama et al. [173] showed that caffeine was not associated with a higher risk of urinary incontinence in a group of 683 men and 298 women (aged 40–75 years) from Japan. On the other hand, Townsend et al. [174], who examined 21,564 women (aged 39–81 years) with moderate urinary incontinence showed that long-term caffeine intake over one year was not associated with urinary incontinence progression over two years among women, either when 150 mg nor 450 mg of caffeine per day were given. In 2013, Gleason et al. [175] examined 4309 non-pregnant women (>20 years old) with urinary incontinence (from mild to severe forms) and showed that caffeine intake ≥204 mg/day was associated with urinary incontinence, but not with severe forms of it.

#### 2.6.2. Caffeine and Kidney Stones

Studies by Curhan et al. [176] showed that there is an association between intake of caffeinated drinks and risk of kidney stones. The authors based their data on the study of 553,081 women, of which 719 had documented kidney stones. They reported that risk for stone formation decreased by 10% for caffeinated coffee and 8% for tea for each 240 mL serving consumed daily. Peerapen and Thongboonkerd [177] in their study investigated the formation of calcium oxalate monohydrate (COM) (which is one of the main causes of kidney stones) in vitro after use of different doses of caffeine (1 to 10 μM). The authors examined the crystallization process, crystal growth, and crystal-cell adhesion, and found that caffeine reduced the crystal number and crystal-binding capacity of renal tubular cells in a dose-dependent manner. The study results showed significantly decreased levels of annexin A1 in the apical surface (a protein that belongs to the annexin family of calcium-dependent phospholipid-binding proteins that plays a significant role in controlling intercellular calcium release), because this protein is translocated into the cytoplasm of the caffeine-treated cells. This mechanism may decrease the crystal-binding capacity of renal tubular epithelial cells [177]. Caffeine has anti-fibrotic activity not only against liver fibrosis, but also against renal fibrosis. Nilnumkhum et al. [178] studied the protective effect of caffeine against renal fibroblast activation induced by hypoxia. The authors used the baby hamster kidney (BHK-21) fibroblast cell line (ATCC) as an in vitro model and inducted hypoxia by transferring them to a hypoxic chamber for 12 h. The control sample were fibroblasts grown under normoxic conditions. Hypoxia increased levels of fibronectin, α-smooth muscle actin, actin stress fibers, intracellular reactive oxygen species (ROS), and oxidized proteins, but caffeine preserved all these markers to their basal levels. Additionally, cellular catalase (CAT) activity (reduced by hypoxia) could be reactivated by caffeine. The authors showed that caffeine eliminated intracellular ROS and therefore exhibited an anti-fibrotic effect against hypoxia-induced renal fibroblast activation [178].

#### 2.6.3. Caffeine and Bladder Cancer

The protective role of caffeinated coffee in bladder cancer is still being considered. Some meta-analysis studies showed no correlation between coffee consumption and risk of bladder cancer [179], but other meta-analyses of case-control studies provide opposing data, suggesting a linear increase in the risk of bladder cancer along with the amount of coffee intake (15–29% increase for 2–4 cups of coffee a day, proportionally) [180]. Moreover, Huanhuan et al. [144] examined the effect of caffeine in the inhibition of renal cell carcinoma (RCC) and showed that caffeine may target glucose-6-phosphate dehydrogenase (G6PDH), inhibit G6PDH activity, and disrupt redox homeostasis, and through this, may inhibit RCC tumor growth, which is dependent on G6PDH activity. This discovery opens the gates to the therapeutic effects of caffeine in kidney diseases, even cancer [181].

In summary, the effect of caffeine on the urinary system may be direct or indirect, and caffeine can act through the products of its metabolism, which are excreted by the kidneys, and this process depends on age and sex. Women are more likely to experience urinary incontinence problems. There are also positive effects such as a reduction in kidney stone formation thanks to a decrease in the crystal-binding capacity of renal tubular epithelial cells, and lower renal fibrosis because of its ability to eliminate ROS. The role of caffeine in bladder cancer is still under investigation, but there are premises that caffeine increases the risk of this ailment.

### 2.7. Skeletal and Muscular System

Caffeine’s capacity to improve exercise performance and cognitive functions makes it a very common dietary supplement in sports nutrition [182]. It is suggested that the most important mechanism of caffeine activity in muscle work is antagonism of ARs. Preventing the decrease in neuronal activity by blocking the ARs is associated with the possibility of increasing muscle fiber recruitment [183]. Another mechanism of the caffeine effect is the opening of the ion channel RyRs, especially in muscles and myocytes [184]. There is a reserve of Ca^2+^ in the sarcoplasmic reticulum (SR), which can be additionally released in the presence of caffeine, resulting in improved muscle speed and strength [185]. Caffeine can increase contractility during submaximal contractions through induction calcium release from SR and inhibition of its reuptake [163]. The ability of caffeine to boost adrenaline rush, release calcium ions, improve Na⁺/K⁺-ATPase, and reduce pain perception [186] seems to be directly related to improved sports performance. Caffeine may also have a direct positive effect on the mechanical activity of skeletal muscle. This was demonstrated by Domaszewski et al. [187], who studied 40 professional male handball players (age: 23.13 ± 3.51 years, body mass: 93.51 ± 15.70 kg, height: 191 ± 7.72 cm, BMI: 25.89 ± 3.10) who regularly consumed products rich in caffeine by giving them caffeine at a dose of 9 mg/kg body weight. The authors observed improved contraction time and reduced maximal displacement in the tested group [187].

#### 2.7.1. Caffeine and Bones

The connection between caffeine and osteogenic activity was examined by Shin et al. [188] in a study on 51 two-week-old male rats. The authors showed that high-caffeine consumption (120 and 180 mg/kg/day) for four weeks led to a significant decrease in body mass gain with proportional decreases in lean body mass and body fat. Additionally, in dual-energy X-ray and F-NaF positron emission tomography, a decrease in bone mass and in vivo osteogenic activity was observed—a shorter and lighter tibia, femur, and vertebral column. Caffeine intake may have a small negative effect on calcium levels, but there is not enough evidence to show an association between coffee consumption and risk of osteoporosis [189].

#### 2.7.2. Caffeine Action on Muscle Filaments and Muscular Strength

Caffeine, an activator of the calcium and cAMP/protein kinase A (cAMP/PKA) pathway, enhances glucose uptake, fat oxidation, and mitochondrial biogenesis in skeletal muscle cells. Yokokawa et al. [190] reported that caffeine may increase myoglobin expression via the cAMP/PKA pathway in skeletal muscle by using L6 myotubes. The authors showed that caffeine increased myoglobin expression and activated the cAMP/PKA pathway in muscle cells. Moreover, cAMP increased myoglobin expression [190]. Tazzeo et al. [191] proved that caffeine decreases actin filament binding to phosphorylated myosin heads and increases the ratio of globular to filamentous actin in pre-contracted tissues, and concluded that caffeine interferes with actin function (decreased binding by myosin, possibly with depolymerization), and for this reason, relaxes smooth muscle [191].

Caffeine in a dose of 3 mg/kg body weight induced changes in muscle oxygen saturation during submaximal workloads [143]. Wilk et al. [192] examined 16 healthy strength-trained male athletes (age: 24.2 ± 4.2 years, body mass: 79.5 ± 8.5 kg, BMI: 24.5 ± 1.9, bench press 1RM: 118.3 ± 14.5 kg), who were habitual caffeine consumers (411 ± 136 mg of caffeine per day). In the study, the athletes’ response to caffeine was examined (9 mg/kg body mass and 11 mg/kg body mass) after strength and muscle endurance tests. The authors showed that the dose of caffeine was associated with peak velocity and reported its significant decrease when 11 mg/kg body weight of caffeine was used, but no other changes were observed. Cesareo et al. [193] in 2019 achieved similar results in their study based on 12 resistance-trained men (aged 20–29 years) and showed that a caffeine-like compound (TeaCrine^®^ 300 mg) with a caffeine content of 300 mg did not improve muscular strength, power, or endurance performance [193]. However, future studies should confirm the above findings and also analyze the inter-subject variations in response to different doses of caffeine.

In conclusion, the influence of caffeine on the functioning of muscular and skeletal systems is important in sport, mainly because of its ability to improve exercise performance and cognitive functions by antagonism of ARs, opening RyRs channels and induction of calcium release. Scientists have focused their attention on athletes to demonstrate or exclude the potential dose-dependent doping nature of caffeine, however, its action should also be analyzed in the context of other strength sports. Regarding the contribution of caffeine to some diseases, it has not been possible to show an effect on the risk of muscle and bone disease (e.g., osteoporosis), but this area of research has still not been completely explored, and a great deal of further research is needed in order to be able to draw more constructive conclusions.

## 3. Caffeine and Oxidative Stress

Oxidative stress is the result of the negative effect of reactive oxygen (ROS) and nitrogen (RNS) species, which, under favorable circumstances, are a cause of the disruption of oxidative–antioxidant balance. ROS and RNS are produced continuously in the human body through oxidative metabolism, mitochondrial bioenergetics, and immune function. Indirectly, nutrients that induce inflammation are also involved in the development and maintenance of oxidative stress. There are different types of sources of nutrient-mediated oxidative stress that play a key role in the development of many human diseases [194]. Caffeine can indicate oxidative stress of varying severity depending on the person’s sex, age, health, body weight, BMI, and lifestyle, and also depending on type, dose, and mode of preparation of coffee [195]. In some studies, caffeine showed concentration-dependent non-enzymatic antioxidant potential by decreasing the levels of free radical generation and reducing superoxide dismutase (SOD) and CAT activities [196]. Caffeine protects against cell damage, exerts antioxidant effects, and reduces oxidative stress markers [197]. Chu et al. [198] investigated the effect of coffee (both caffeinated and decaffeinated) on oxidative stress induction by using primary neuronal cell culture and H_2_O_2_ and showed that the high levels of chlorogenic acid lactones (CGLs) and lipophilic antioxidants included in roasted coffee could protect neuronal cells against the oxidative stress induced by H_2_O_2_ by modulation of the ERK1/2 (extracellular signal-regulated kinases 1/2) and JNK (c-Jun N-terminal kinases) signaling pathways. Moreover, the authors indicated that only the roasted coffee extract inhibited JNK activation, while both roasted and green coffees inhibited ERK1/2 activation and showed a neuroprotective effect on neurodegenerative processes [198].

Li et al. [199] examined the role of caffeine in skin protection. Their study showed that a low dose of caffeine (<10 μM) could suppress skin damage induced by dihydrochloride or ultraviolet, which they used to induce oxidative stress in transformed skin cells and normal human epidermal keratinocytes. Caffeine can also mechanistically facilitate the elimination of ROS by activating autophagy through the inhibition of AR, increasing the level of sirtuin 3 (SIRT3) and the activation of 5′ adenosine monophosphate-activated protein kinase [199]. Similar investigations were also conducted by Xu et al. [200], and the authors showed that caffeine (4–512 μM) promoted SIRT3 activity and reduced SOD2 (superoxide dismutase 2) acetylation. The results of the above studies show that caffeine targets SIRT3 to enhance SOD2 activity and protect skin cells from UV irradiation-induced oxidative stress. Thus caffeine, as a small-molecule SIRT3 activator, could be a potential agent to protect human skin against UV radiation [200].

In summary, it should be emphasized that caffeine, acting as an antioxidant, counteracts the negative effects of oxidative stress and oxidative–antioxidant imbalances. Caffeine, by decreasing the levels of free radical generation, protects neuronal cells against oxidative stress and damage, and has a neuroprotective effect on neurodegenerative processes. Additionally, caffeine can suppress skin damage induced by ultraviolet and can mechanistically facilitate the elimination of ROS, and thus protect skin cells from UV irradiation-induced oxidative stress. The proven protective function of caffeine against the harmful effects of UV radiation and the free radicals generated by it on the skin is an interesting topic for future research including studies aimed at using scientific discoveries not only in pharmacology, but also in cosmetology.

## 4. The Association between Caffeine Consumption and All-Cause and Cause-Specific Mortality

Caffeine consumption may be associated with positive health influence, but also with side effects. The worst case scenario directly related to the consumption of gram amounts of caffeine is death, usually in attempted suicide, although in these cases, it is also rare. The increased risk of adverse effects of caffeine should be considered in poor metabolizers, people with liver disease, and people with heart disease, who may die from ingesting caffeine at levels well below what is normally considered toxic [32].

In normal condition, caffeine is supposed to be associated with lower risk of death. The mechanism by which it may reduce mortality are not well-known, but it is suggested that it could be due to its antioxidant and anti-inflammatory effects [201]. Tsujimoto et al. [202] conducted a prospective cohort study based on the data from the National Health and Nutrition Examination Survey 1999–2010 of about 17,594 participants. The authors compared hazard ratios (HR) for death among participants with a caffeine intake of 10–99, 100–199 and 200 mg/day or more with those whose caffeine consumption was less than 10 mg/day. In their main analysis, caffeine intake was associated with lower risk of all-cause mortality. They also performed an additional analysis among patients with diabetes and reported a non-significant association between caffeine intake and mortality among participants with this disease, but this conclusion was challenged by Neves et al. [203], who, after taking into account sex, presence of kidney disease, and dietary habits of participants, showed a dose-dependent protective effect of caffeine intake among women with diabetes and may reduce their mortality. An inverse relationship between caffeine consumption and all-cause mortality has also been reported among people with chronic kidney disease (CKD) by Vieira et al. [204]. The authors analyzed 4863 adults with CKD and showed that patients who consumed >213.5 mg/day of caffeine were less exposed to death from CKD than those who consumed <28.2, 28.2–103.0, and 103.01–213.5 mg/day of this purine alkaloid. In a meta-analysis conducted by Li et al. [205], composed of 21 cohort studies, the consumption of caffeinated coffee was also associated with decreased all-cause mortality. 

Caffeine is widely viewed as a safe stimulant, and is consumed in a variety of forms from pure caffeine to caffeinated drinks and food. However, it should be noted that excessive caffeine consumption may be hidden. For example, herbal medicines and herbal supplements for weight loss with high caffeine content may not disclose these concentrations on the product label, which may be an underestimated source of caffeine in this context. In addition, interactions between caffeine and other ingredients in caffeine-containing products may also increase the risk of side effects [32]. Nevertheless, caffeine intake is associated with lower risk of all-cause mortality and for this reason may be useful in life extension, especially among women with diabetes and patients with CKD. These results lead to the promising consideration of the use of caffeine as an agent in the treatment of many diseases and should be taken into consideration in further research, not forgetting the negative effects of its activity.

## 5. Conclusions

It is not easy to define unequivocally whether caffeine has a positive or negative effect on the human organism. Through interaction with receptors such as ARs, RyRs, and GABA receptors and inhibition of PDEs, caffeine’s action is multi-directional and reaches most of the human body systems. Its prevalence in plants, medicinal and other products means that most people are exposed to its use, and its rapid absorption and complex metabolism contribute to its effects on cell function. Caffeine’s action depends on age, sex, source, and consumed dose. At low doses, caffeine is said to have a positive effect on cognitive performance, memory, and brain function, but at high doses, it may be responsible for nervousness, anxiety etc. The positive effects of caffeine are observed in many diseases (AD, PD, asthma, cirrhosis, fibrogenesis, kidney stones, some cancers, etc.) as well as negative effects (Huntington’s disease, arrythmia, tachycardia, lung cancer etc.). Caffeine is also considered to have a therapeutic impact on pain. Thanks to its anti-inflammatory and antioxidant properties, it may be useful medication not only in pharmacology, but also in cosmetology. Many studies have focused on caffeine’s ability to improve motor and respiratory functions, which seems to be important in sport. The multi-directional action of caffeine is a very interesting research direction, and therefore this topic was, is, and will continue to be investigated, which will contribute to the development of new medicines, increasing social awareness of the effects of caffeine consumption, and constantly expanding knowledge about this commonly used stimulant.

## Figures and Tables

**Table 1:** The main effects of caffeine action on the nervous system and the most likely mechanisms of its action.

Disease/Disorder	Mechanism	Effect
Alzheimer’s disease	↓ Aβ generation, activation of ARs, antioxidant activity	↓ risk of disease [45,50,97]
Parkinson’s disease	Activation of ARs	↓ risk of disease [47,51,98]
Huntington’s disease	Unknown	↑ risk of disease [57]
Depression	Antagonism of ARs	↓ risk of disease [45,96,102]
Mood	Antagonism of ARs, inhibition of AChE, impact on RyRs	stimulation at low doses [8,27] ↑ restlessness, excitement, tremor, tinnitus, headache, and insomnia at high doses [30,34] ↑ anxiety [94,95,96]

Aβ—amyloid beta, Ars—adenosine receptors, AChE—acetylcholinesterase, RyRs—ryanodine receptors. ↓—decrease, ↑—increase.

**Table 2:** The effects of caffeine action in respiratory system diseases.

Disease	Effect
Breathing problems	↑ ventilation [150,151] ↑ tidal volume [150,151] ↑ inspiratory flow [150] ↓ total lung resistance [151]
Asthma COPD	↑ airways functions [146] ↑ risk of COPD [147]
Lung cancer	↑ risk of lung cancer [152]

COPD—Chronic Obstructive Pulmonary Disease. ↓—decrease, ↑—increase.

## Data Availability

Not applicable.
